# Healing Rate and Clinical Outcomes of Lesser Tuberosity Osteotomy for Anatomic Shoulder Arthroplasty

**DOI:** 10.5435/JAAOSGlobal-D-19-00119

**Published:** 2020-02-10

**Authors:** Nick R. Johnson, David P. Trofa, Bryan M. Saltzman, Katherine R. Muña, Shadley C. Schiffern, Nady Hamid

**Affiliations:** From the Department of Orthopaedic Surgery (Dr. Johnson), Carolinas Medical Center; the OrthoCarolina Shoulder and Elbow Center (Dr. Trofa, Dr. Saltzman, Dr. Schiffern, and Dr. Hamid); and the OrthoCarolina Research Institute (Dr. Muña), Charlotte, NC.

## Abstract

Several techniques are available for subscapularis management during total shoulder arthroplasty (TSA). Lesser tuberosity osteotomy (LTO) is advocated owing to improved biomechanical strength and high rates of healing. However, displacement or nonunion of the LTO could theoretically cause functional deficits for the affected shoulder. The purpose of this study is to examine the healing rate and clinical outcomes of LTO in patients undergoing anatomic TSA.

**Methods:**

A retrospective review of our institution's prospectively enrolled TSA registry was performed. Patients undergoing primary TSA who had an LTO performed and radiographs at a minimum of 3 months postoperatively were included. Two fellowship-trained shoulder and elbow surgeons reviewed all radiographs and categorized LTO healing into three groups: healed, nondisplaced nonunion, or displaced nonunion. Physical examination data and patient-reported outcome scores including American Shoulder and Elbow Score, Single Assessment Numeric Evaluation scores, and the Veteran Rand mental and physical component scores were obtained at a median of 1 year after surgery. Comparative statistical analysis was performed on the healed versus nondisplaced nonunion groups.

**Results:**

We included 142 shoulders in 130 patients who met the inclusion criteria with an average age of 65.2 years (SD: 10.3). Radiographic evaluation at a median of 1.0 years postoperatively (range: 6 months to 2.2 years) revealed 124 (87%) healed LTO, 12 (8%) nondisplaced nonunions, and six (5%) displaced nonunions. The median American Shoulder and Elbow Score total score was 89.2 (IQR: 72.2, 98.3) in the healed LTO group versus 96.7 (30, 98.3) in the LTO nondisplaced nonunion group (*P* = 0.9637). The median Veteran Rand mental component was 55.1 (IQR: 43.4, 61.0) in the healed LTO group versus 54.6 (38.8, 58.2) in the LTO nondisplaced nonunion group (*P* = 0.5679). The median Single Assessment Numeric Evaluation score was 85.0 (IQR: 70.0, 95.0) in the LTO-healed group versus 75.1 (35.0, 97.1) in the LTO nondisplaced nonunion group (*P* = 0.7699). There were no significant differences in revision surgery occurrence between the groups to address subscapularis instability: one patient in the LTO-healed group underwent revision surgery for subscapularis repair 3 months after primary surgery because of continued pain and weakness, and no patients in the other LTO groups underwent revision surgery.

**Conclusion:**

Although there is a risk of nonunion and displacement using the LTO technique in TSA, the overall clinical outcomes and radiographic union rates are high with a very low risk of revision surgery. In addition, radiographic evidence of nonunion does not significantly correlate with clinical outcomes.

Mobilization and repair of the subscapularis is most often used during total shoulder arthroplasty (TSA). Failure of the subscapularis repair remains a common problem after TSA and can occur secondarily to many factors including excessive tension because of oversized components, less than optimal tissue, aggressive rehabilitation protocol, and nerve injury during mobilization of the subscapularis tendon.^1,2,3,4^ Several techniques are available for subscapularis management during TSA. Historically, the subscapularis has been treated with tenotomy medial to the insertion of the tendon off the lesser tuberosity. However, this technique was debated after reports of complications including abnormal lift-off and belly-press tests and subscapularis rupture with anterior instability.^1,5,6^

To address difficulties with soft-tissue approaches, the lesser tuberosity osteotomy (LTO) was described as an alternative in which the tendon is detached with a piece of bone.^7^ This technique provides bone-to-bone healing which is theoretically thought to be more reliable than tendon-to-tendon healing and is advocated because of the reports of improved biomechanical strength and high rates of healing.^5,8,9,10,11^ However, displacement or nonunion of the LTO could theoretically cause functional deficits for the affected shoulder. Recent studies have called into question the superiority in results achieved using LTO and highlighted the negative aspects of the technique, including increased operative time, potential for proximal humeral fracture, and compromise of press-fit short-stem fixation.^12,13^ Recent biomechanical studies have found equivocal results and less variability in strength using subscapularis peel compared with LTO.^14^ In addition, recent randomized controlled trials have found equivalent clinical results.^13,15,16^

The aim of this study is to examine the healing rate and clinical outcomes of LTO in patients undergoing anatomic TSA. It was hypothesized that LTO would provide reliable healing, good functional outcomes for patients, and low need for revision surgery.

## Methods

**Figure 1:**
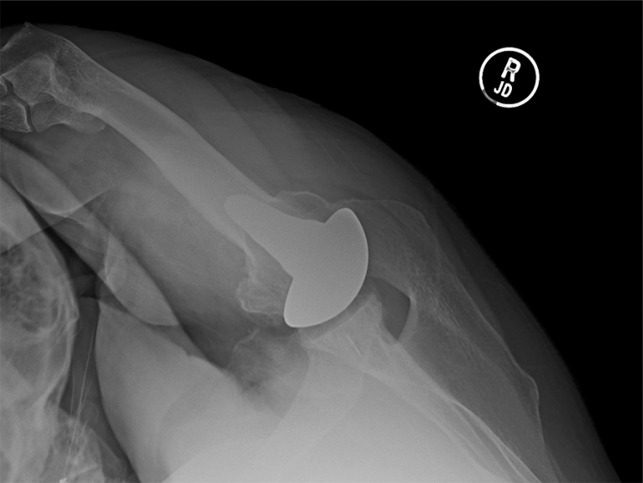
Radiograph showing the example of a healed, union of a lesser tuberosity osteotomy.

**Figure 2:**
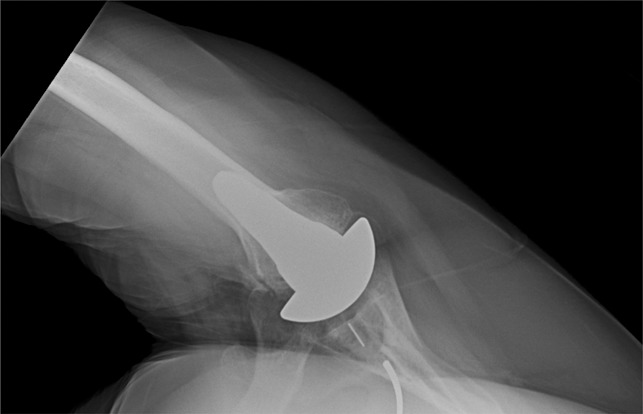
Radiograph showing the example of a nondisplaced, nonunion of a lesser tuberosity osteotomy.

**Figure 3:**
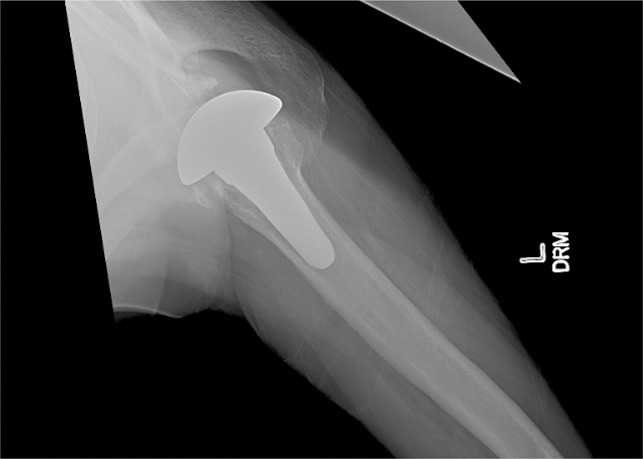
Radiograph showing the example of a displaced, nonunion of a lesser tuberosity osteotomy.

A retrospective review of our institution's prospectively enrolled TSA registry was performed. Patients undergoing primary TSA who had an LTO performed and radiographs at a minimum of 3 months postoperatively were included. Two fellowship-trained shoulder and elbow surgeons reviewed all radiographs and categorized LTO healing into three groups: healed, nondisplaced nonunion, or displaced nonunion. Sequential postoperative radiographs were reviewed using AP, scapular Y, and axillary views were used to evaluate the LTO fragment. Images were classified as a union, a nondisplaced, nonunion and displaced, nonunion as described by Small et al^17^ (Figures 1–3). Physical examination data, including internal rotation, external rotation, forward flexion, and patient-reported outcome scores including American Shoulder and Elbow Score (ASES), Single Assessment Numeric Evaluation (SANE) scores, and the Veteran Rand (VR-12) mental and physical component scores were obtained at a median of 1 year after surgery. A comparative statistical analysis was performed on the healed versus nondisplaced nonunion groups.

The technique used for LTO was similar to the previously described techniques,^18^ and it entailed creating an osteotomy beginning at the bicipital groove extending from the rotator interval superiorly to the circumflex vessels inferiorly. Any remaining articular surface was removed from the bone fragment before repair. The LTO repair was performed with nonabsorbable suture using the transosseous bone tunnels, and the sutures are passed medial to the osteotomy because the sutures are going through the subscapularis and passing the suture through the tendinous portion of the subscapularis. Rotator interval closure was also performed to augment the LTO repair.

Supervised physical therapy was begun immediately after surgery. The rehabilitation protocol for patients postoperatively called for 4 weeks of sling immobilization with passive range-of-motion (ROM) exercises that began immediately after surgery. Active ROM with sling discontinuation began after 4 weeks. Strengthening exercises began after 12 weeks with full return to the recreational activities at 6 months postoperatively.

Study data were collected and managed using REDCap electronic data capture tools hosted at the OrthoCarolina Research Institute.^19^ All data underwent statistical analysis using SAS version 9.4 (SAS Institute, Cary, NC; http://www.sas.com/software/sas9). Descriptive statistics were produced and chi-square tests (or Fisher exact tests, where appropriate) for categorical or Wilcoxon two-sample tests for continuous non-normally distributed outcomes were used for comparisons between groups. Inter-rater reliability was assessed on radiographic outcomes using the prevalence- and bias-adjusted Kappa statistic.

## Results

We included 142 shoulders in 130 patients who met the inclusion criteria with an average age at surgery of 65.2 years (SD: 10.3) and Body Mass Index of 30.9 (7.4). Sixty-six (50.8%) patients were men, and 74 (56.9%) never smoked (Table 1).

**Table 1:** Demographics

Patient Demographics	N = 130 Patients
Sex, n (%)	
Men	66 (50.8%)
Women	64 (49.2%)
Age (yrs) at surgery, mean (SD)	65.2 (10.3)
BMI (kg/m^2^) at surgery, mean (SD) (missing for 1 patient)	30.9 (7.4)
Race, n (%)	
Caucasian	111 (85.4%)
Black, African American	17 (13.1%)
Unknown	2 (1.5%)
Ethnicity, n (%)	
Not Hispanic or Latino	122 (93.8%)
Hispanic or Latino	1 (0.8%)
Declined	7 (5.4%)
Smoking, n (%) (missing for 2 patients)	
Never	74 (56.9%)
Former	50 (38.5%)
Current	4 (3.1%)

BMI = body mass index

Of the 142 shoulders radiographically reviewed at a median of 1.0 years postoperatively (range, 6 months to 2.2 years), there were 124 (87%) healed LTO, 12 (8%) nondisplaced nonunions, and 6 (5%) displaced nonunions. There was strong inter-rater reliability between our two radiographic readers resulting in a 0.98 prevalence- and bias-adjusted Kappa statistic (95% confidence interval: 0.95, 1.0). Six patients (4.1%) experienced a complication where three sustained fractures about the proximal humerus and three sustained other complications including component loosening, rotator cuff tear, and chronic pain at an average of 2.0 years after surgery. Two patients required reoperations; one required revision subscapularis repair 3 months after primary TSA and the other patient required arthroscopic evaluation for chronic pain at 1.4 years after primary TSA. No patients had radiographic evidence or clinical reports of dislocation or infection at the final follow-up.

Postoperative ROM was found to be similar between healed LTO and nonunion nondisplaced groups with no difference in the internal rotation (*P* = 0.6255).

Patient reported outcomes (PROs) were collected at a median of 1.0 year (range, 6 months to 2.2 years) postoperatively. Overall, the median ASES total score was 90.0 (Interquartile Range [IQR]: 73.3, 98.3), and median VR-12 physical and mental component scores were 43.0 (IQR: 34.0, 50.7) and 55.0 (43.6, 60.7), respectively. The median SANE score was 85.1 (Table 2). When stratified based on the radiographic outcomes as healed LTO vs. LTO nondisplaced nonunion, no statistically significant differences in patient-reported outcomes were found. The median ASES total score was 89.2 in the healed LTO group versus 96.7 in the LTO nondisplaced nonunion group (*P* = 0.9637). The median VR-12 mental component was 55.1 in the LTO-healed group versus 54.6 in the LTO nondisplaced nonunion group (*P* = 0.5679). Finally, the median SANE score was 85.0 in the LTO-healed group versus 75.1 in the LTO nondisplaced nonunion group (*P* = 0.7699) (Tables 3 and 4).

**Table 2:** Patient-reported Outcomes

PRO Variables	Overall (N = 130 Possible)
ASES (patient), median (IQR) (n)	
VAS pain (0–10)	0.3 (0.0, 3)
Function subscore	43.3 (33.3, 48.3)
ASES total score	90 (73.3, 98.3)
VR-12, median (IQR) (n)	
Physical component score	43 (34, 50.7)
Mental component score	55 (43.6, 60.7
SANE score, median (IQR) (n)	85.1 (66.9, 95.1)

ASEA = American Shoulder and Elbow Score, SANE = Single Assessment Numeric Evaluation, VR-12 = Veteran Rand

**Table 3:** Range of Motion and Patient-reported Outcomes (Stratified by Group)

ROM Variables—Shoulder level	Stratified by Group		*P* Value^a^	Displaced (N = 6 Shoulders)
	Healed (N = 124)	Nonunion/Nondisplaced (N = 12)		
Active forward elevation (degrees), median (IQR)	160 (150, 167.5)	150 (140, 165)	0.5253	150 (140, 150)
External rotation, median (IQR)	45 (45, 65)	55 (37.5, 70)	0.9686	47.5 (45, 65)
Internal rotation, n (%)			0.6255	
Poor (<T12)	47 (37.9%)	6 (50.0%)		3 (50.0%)
Acceptable (T12-T9)	40 (32.3%)	4 (33.3%)		1 (16.7%)
Normal (>T9)	18 (14.5%)	0		0

PRO Variables—Patient Level^b^	Stratified by Group		*P* Value^a^	Displaced (N = 6 patients)
	Healed (N = 113 patients)	Nonunion/Nondisplaced (N = 11 patients)		
ASES (patient), median (IQR^c^) (n)				
VAS pain (0–10)	0.5 (0, 3)	2.4 (0, 7.3)	0.5244	0 (0, 0)
Function subscore	43.3 (33.3, 48.3)	48.3 (15, 50)	0.5114	37.5 (33.3, 41.7)
ASES total score	89.2 (72.2, 98.3)	96.7 (30, 98.3)	0.9637	87.5 (83.3, 91.7)
VR-12, median (IQR^c^) (n)				
Physical component score	43 (34, 50.7)	44 (32.6, 49.7)	0.9251	45 (38.4, 51.6)
Mental component score	55.1 (43.4, 61)	54.6 (38.8, 58.2)	0.5679	49.1 (46.2, 52.1)
SANE score, median (IQR^c^) (n)	85 (70, 95)	75.1 (35, 97.1)	0.7699	85.2

ASES = American Shoulder and Elbow Score, LTO = lesser tuberosity osteotomy, SANE = Single Assessment Numeric Evaluation, ROM = range of motion, VR-12 = Veteran Rand

a Statistical tests performed on healed vs. nonunion nondisplaced groups. Displaced group were included in the table for descriptive purposes. Chi-square or Fisher exact tests were used for categorical data and Wilcoxon rank-sum tests were used for continuous non-normally distributed data to determine statistical significance between groups at an alpha level of 0.05.

c The IQR within parentheses is also the range for the displaced LTO group because of the low sample sizes of completed PROs.

b The first surgery occurrence was used for the patient specific data and associated comparative analyses.

**Table 4:** Sub-analysis ROM (Stratified by Group)

ROM Variables—Shoulder Level	Stratified by Group		Displaced (N = 6)	*P* Value^a^
	Healed (N = 124)	Nonunion/Nondisplaced (N = 12)		
Active forward elevation (degrees), median (IQR)	160 (150, 167.5)	150 (140, 165)	150 (140, 150)	0.6447
External rotation, median (IQR)	45 (45, 65)	55 (37.5, 70)	47.5 (45, 65)	0.3052
Internal rotation, n (%)				0.9603
Poor (<T12)	47 (37.9%)	6 (50.0%)	3 (50.0%)	
Acceptable (T12-T9)	40 (32.3%)	4 (33.3%)	1 (16.7%)	
Normal (>T9)	18 (14.5%)	0	0	

ROM = range of motion

a Chi-square or Fisher exact tests were used for categorical data and Kruskal-Wallis tests were used for continuous non-normally distributed data to determine statistical significance between groups at an alpha level of 0.05.

## Discussion

The current study reinforces what has been seen in previous studies—LTO as a method of mobilization of the subscapularis tendon during TSA and is safe and effective. Patients in the current study achieved good clinical and functional outcomes, low needs for revision surgery, and satisfactory results. The key role the subscapularis plays in maintaining anterior shoulder stability has repeatedly been demonstrated throughout the literature.^20,21^ Preventing subscapularis dysfunction is of key importance after TSA. Subscapularis dysfunction has repeatedly been associated with pain, dysfunction, and need for further operations after TSA.^1,2^ The goal of this study was to show the efficacy and safety of LTO for subscapularis management after primary TSA. We found that 87% of the cohort had healed LTO at the final radiographic follow-up. Although some other studies have reviewed CT versus the current study which evaluated the plain radiographs, our findings are similar to the previous cohorts. Lapner et al^15^ found similar healing rates at the final follow-up where LTO healed at a 95% rate at the final CT follow-up. Other high-level studies, including a randomized controlled trial and a large radiographic review, demonstrated similar healing rates to our study that were greater than 85% union at the final follow-up.^17,18^ This high rate of healing makes this technique desirable when compared with other tendon-to-tendon healing techniques. LTO preserves the integrity of the subscapularis. The proximal humerus has a high potential for healing, and tendons have shown to have a low potential for healing in older patients, such as those in our cohort.^22,23^ LTO provides reliable healing rates, which was proven in our study.

We did not find a significant difference in clinical and functional outcomes in our cohort between patients who had a united LTO versus those who had a nondisplaced nonunion of their LTO on final radiographs. This finding was surprising because subscapularis deficiency is a well-known and common cause of TSA failure. This may be because of the low numbers of patients in the nonunion group or the lack of sensitivity of the patient-reported outcome tools to detect a subtle difference between these groups. It also remains to be seen if a longer follow-up will show a difference in the clinical outcomes in this cohort. In a recent study, Levy et al^24^ reviewed a cohort of 189 patients and examined how radiographic healing impacted the outcomes. They elucidated that patients with united LTO and nondisplaced LTOs had better postoperative simple shoulder test scores, ASES Scores, and VAS scores compared with those with displaced nonunions.

Based on our clinical experience and the data from the current study agree with this statement because in our cohort the few displaced, nonunion patients had worse outcomes; however, given the low sample size of the displaced group, we were unable to validly prove this statistically. They also found more improvement in these scores compared with preoperative scores in those with united LTO and nondisplaced nonunion LTOs. Although they did find a difference in the clinical outcome scores, they found, as we did, no difference in overall satisfaction.

The literature is still not clear whether one method of subscapularis management in TSA is superior. In a recent meta-analysis, Choate et al. found two studies that included postoperative ASES scores after LTO, averaging 73 at the final follow-up. These were compared with subscapularis tenotomy and peel provided scores of 80.8 and 79.1, respectively. No analysis was given on whether this was a statistical difference; however, it does not fall within the minimal clinically important difference in ASES scores that has been previously reported in the shoulder arthroplasty literature.^25^ This was a meta-analysis that included studies from the early 2000s. Our difference may be attributed to improved strategies in using the LTO technique since its description in 2005. This is demonstrated in a recent study by Aibinder et al^26^ who found similar ASES scores in patients who underwent LTO during TSA to the current cohort. Their study found the ASES scores of 89 after LTO, which is very similar to the current cohort. This supports the hypothesis that as the technique has evolved surgeons have learned how to better use it to maximize healing and improve outcomes. The current study is consistent with previous studies in that LTO provides very satisfactory patient-reported outcomes.

Several strengths of the current study are present. It includes a large cohort with a good midterm follow-up. The current study's cohort is one of the largest than that has been examined in the literature. Patient-reported outcome scores and clinical examination data were able to be collected using a prospectively collected database. It included patients from only two fellowship-trained shoulder surgeons. However, the current study does have several limitations. First, there is no control group using other methods of mobilization for the subscapularis tendon. In addition, there were no CT scans performed to evaluate union at the final follow-up which provide a better evaluation of union after LTO. We also did not have data on lift-off test or belly-press test which has been shown to be the best way to assess subscapularis function by physical examination.^27,28^

## Conclusion

In summary, LTO provides a safe and effective method in mobilizing the subscapularis during TSA. A risk of nonunion and displacement using the LTO technique is present, but overall clinical outcomes and patient satisfaction remained high. Anecdotally, we believe our results show that those with displaced, nonunited lesser tuberosities have worse outcomes; however, this was unable to be statistically confirmed in our manuscript because of low sample size. In addition, radiographic evidence of nonunion did not correlate with the inferior clinical outcomes.
